# Planar polarization of Vangl2 in the vertebrate neural plate is controlled by Wnt and Myosin II signaling

**DOI:** 10.1242/bio.201511676

**Published:** 2015-04-24

**Authors:** Olga Ossipova, Kyeongmi Kim, Sergei Y. Sokol

**Affiliations:** Department of Developmental and Regenerative Biology, Icahn School of Medicine at Mount Sinai, New York, NY 10029, USA

**Keywords:** Planar cell polarity, Vangl2, Prickle, Myosin II, *Xenopus*, Neural tube closure, Morphogenesis

## Abstract

The vertebrate neural tube forms as a result of complex morphogenetic movements, which require the functions of several core planar cell polarity (PCP) proteins, including Vangl2 and Prickle. Despite the importance of these proteins for neurulation, their subcellular localization and the mode of action have remained largely unknown. Here we describe the anteroposterior planar cell polarity (AP-PCP) of the cells in the *Xenopus* neural plate. At the neural midline, the Vangl2 protein is enriched at anterior cell edges and that this localization is directed by Prickle, a Vangl2-interacting protein. Our further analysis is consistent with the model, in which Vangl2 AP-PCP is established in the neural plate as a consequence of Wnt-dependent phosphorylation. Additionally, we uncover feedback regulation of Vangl2 polarity by Myosin II, reiterating a role for mechanical forces in PCP. These observations indicate that both Wnt signaling and Myosin II activity regulate cell polarity and cell behaviors during vertebrate neurulation.

## INTRODUCTION

Vertebrate neurulation is a complex process that involves biochemical signaling and mechanical forces, originating not only from the cells composing the neural plate, but also from the neighboring epidermis and the underlying mesoderm. At the beginning of neurulation, dorsal convergence causes the tissue to narrow mediolaterally, while extending along the anteroposterior axis (Colas and Schoenwolf, 2001; Davidson and Keller, 1999). Apical constriction along the mediolateral axis of the neural plate promotes its bilateral folding (Sawyer et al., 2010; Schroeder, 1970; Suzuki et al., 2012). Neurulation is completed with the dorsal fusion of the neural folds, radial thinning and rostrocaudal elongation of the neural tube (Colas and Schoenwolf, 2001; Davidson and Keller, 1999; Keller et al., 1992). Due to the complexity of these morphogenetic movements, several hundred genes were implicated in neural tube closure in mouse genetic models (Harris and Juriloff, 2007). These genes encode transcription factors, cell adhesion components, secreted ligands and transmembrane receptors that belong to a number of signaling pathways.

One of the pathways, which have been linked to vertebrate neurulation, is known as the planar cell polarity (PCP) pathway. The main PCP components Van Gogh/Strabismus, Prickle, Frizzled, and Dishevelled were originally identified in *Drosophila* epithelia and shown to form mutually exclusive protein complexes that are localized to opposite sides of each epithelial cell (Devenport, 2014; Peng and Axelrod, 2012; Simons and Mlodzik, 2008; Wang and Nathans, 2007). These complexes organize the cytoskeleton to establish cell and tissue polarity, although some PCP proteins, such as Frizzled and Dishevelled, also function to transduce Wnt signals. Planar polarization of specific PCP components has been also demonstrated in the ascidian mesoderm (Jiang et al., 2005; Shindo and Wallingford, 2014), zebrafish early embryo (Ciruna et al., 2006; Yin et al., 2008), the mammalian cochlea (Montcouquiol et al., 2003), the mouse skin (Devenport and Fuchs, 2008) and the node (Antic et al., 2010; Borovina et al., 2010; Hashimoto et al., 2010; Mahaffey et al., 2013; Song et al., 2010). Genetic inactivation of PCP genes in mouse embryos and loss-of-function studies in lower vertebrate models revealed severe neural tube closure defects (Curtin et al., 2003; Darken et al., 2002; Etheridge et al., 2008; Goto and Keller, 2002; Gray et al., 2011; Lu et al., 2004; Sokol, 1996; Takeuchi et al., 2003; Torban et al., 2008; Wallingford et al., 2013). Although PCP proteins are essential for neurulation, the underlying molecular mechanisms and the specific contribution of Wnt ligands to PCP remain poorly understood.

PCP-dependent neural tube defects are commonly thought to arise from the inhibition of convergent extension movements, which lead to neural tube elongation (Gray et al., 2011; Keller et al., 1992; Tada and Heisenberg, 2012; Wallingford et al., 2013). Alternatively, these defects may be caused by lack of apical constriction, a cell behavior that is essential for neural plate bending along the mediolateral axis (Ossipova et al., 2014; Sawyer et al., 2010; Schroeder, 1970; Suzuki et al., 2012). To better understand the signaling pathways leading to neural tube formation, it is important to know the distribution of various molecular players and the cellular targets of this putative signaling pathway. In the zebrafish neural and mesodermal progenitors, overexpressed *Drosophila* Prickle-GFP construct is localized anteriorly, whereas Dishevelled-GFP appears to be biased towards the posterior side of each cell (Ciruna et al., 2006; Yin et al., 2008). By contrast, the recycling endosome marker Rab11, the exocyst component Sec15, and the PCP protein Diversin/Ankrd6 (Schwarz-Romond et al., 2002) are enriched at the medial side of apically constricting cells along the mediolateral axis of the *Xenopus* neural plate (Ossipova et al., 2014). A recent study revealed a non-homogeneous distribution of several PCP components, including Celsr1, Dvl2 and PDZ-RhoGEF, to the anteroposterior faces of each cell in the chick neural midline (Nishimura et al., 2012). Although it is still unknown which side of the cell these proteins associate with, the authors proposed that PCP proteins stimulate actomyosin contractility along the constricting cell junctions leading to neural tube closure (Nishimura et al., 2012). Further analysis of PCP protein localization in the neural plate is therefore essential to define molecules regulating neural tube formation along both mediolateral and anteroposterior directions.

This study was initiated to gain additional insights into the regulation of PCP in the neural plate. Our immunostaining experiments revealed the polarization of endogenous Vangl2 to the anterior edge of each cell in the neural plate. This anteroposterior planar cell polarity (AP-PCP) was instructed by Prickle and required Wnt-dependent Vangl2 phosphorylation. Strikingly, changes in the activity of Myosin II, a mediator of many morphogenetic processes, also affected the Vangl2 polarity. We propose that AP-PCP is a conserved feature of chordate embryos that is maintained by both Wnt signaling and mechanical forces.

## MATERIALS AND METHODS

### *Xenopus* embryo culture and microinjections

*In vitro* fertilization and culture of *Xenopus laevis* embryos were carried out as previously described (Dollar et al., 2005). Staging was according to Nieuwkoop and Faber (Nieuwkoop and Faber, 1967). For microinjections, four- to eight-cell embryos were transferred into 2% Ficoll in 0.3× MMR buffer and 5 nl of mRNAs or MO solution was injected into one or more blastomeres. Amounts of injected mRNA per embryo have been optimized in preliminary dose-response experiments (data not shown) and are indicated in figure legends. Vangl2 protein localization defects were scored as the relative numbers of cells with the anterior Vangl2, based on two to five independent experiments. Each experimental group contained 5–10 embryos.

Animal care and use was in accordance with the guidelines established by the Icahn School of Medicine at Mount Sinai, New York.

### Plasmids, mRNA synthesis, morpholinos

The plasmids encoding mouse HA-Vangl2 and HA-Vangl2SA (S5A/S82A/S84A) (Gao et al., 2011), *Xenopus* CFP-Vangl2/Stbm, Wnt5a (Itoh et al., 2009), YFP-DmPk, zPk1ΔPL and GFP-CAAX (Ossipova et al., 2014), DN-Wnt11 (Tada and Smith, 2000), DN-Wnt5a (Choi and Sokol, 2009), DN-ROCK (Marlow et al., 2002), Mypt1 T696A (Weiser et al., 2009), mRFP and *LacZ* (Itoh et al., 2014). Zebrafish Pk1 plasmid was a gift from T. Masa. The PET and LIM domains were removed in zPk1ΔPL mutant by PCR-based mutagenesis. Details of cloning are available upon request.

Capped mRNAs were made by *in vitro* transcription from the T7 or SP6 promoters using mMessage mMachine kit (Ambion). Cytoplasmic GFP (50–100 pg), GFP-CAAX (100 pg) or membrane RFP (100 pg) RNA was coinjected with MOs or RNAs as a lineage tracer. Vangl2/Stbm MO has been described previously (Darken et al., 2002).

### Immunofluorescence and immunoblot analysis

Polyclonal Vangl2 antibodies were generated by immunizing rabbits with the peptide corresponding to amino acids 56–70 of *Xenopus* Vangl2 and affinity purified by binding to the immunogen using standard techniques (Ossipova et al., 2015). Antibodies against the following antigens were used: Vangl2 (1:100, rabbit polyclonal), ZO1 (1:200, Invitrogen, mouse monoclonal), HA (1:200, 12CA5 mouse monoclonal, and 1:3000, polyclonal, Bethyl Labs), GFP (1:200, B-2, Santa Cruz, mouse monoclonal or Invitrogen, rabbit polyclonal). Secondary antibodies were against mouse or rabbit IgG conjugated to Alexa Fluor488, Alexa Fluor555 (1:100, Invitrogen) or Cy3 (1:100, Jackson ImmunoResearch). Standard specificity controls were performed to confirm lack of cross-reactivity and no staining without primary antibodies. Immunofluorescence images were captured using the Axioimager fluorescence microscope (Zeiss) and the Axiovision imaging software (Zeiss). Results shown are representative images from two to five independent experiments, each containing 10–20 embryos per group.

For *en face* immunofluorescent detection of exogenous Vangl2 in the neural plate, early neurula embryos injected with RNAs were devitellinized, fixed for 1 h with MEMFA (0.1 M MOPS, pH 7.4, 2 mM EGTA, 1 mM MgSO_4_ and 3.7% formaldehyde), washed with phosphate buffered saline (PBS) and the neural plate explants were excised and subjected to whole-mount immunostaining. Samples were blocked with 10% goat serum in PBS for 1 h at room temperature, and incubated with anti-HA antibody (Bethyl Labs, 1:3000) at 4°C overnight. Alternatively, for the detection of endogenous Vangl2, embryos were devitellinized, fixed with 2% trichloracetic acid (TCA) solution for 30 min at room temperature, washed with 0.3% Triton X100 in PBS for 30 min (Nandadasa et al., 2009) and stained as described above. For F-actin visualization, MEMFA-fixed embryos were stained with Alexa 568-conjugated phalloidin (5 units/ml, Molecular Probes) overnight at 4°C in 10% goat serum in PBS. After whole mount staining, neural plate explants were dissected with a razor blade and mounted for observation in the Vectashield mounting medium (Vector).

Western blot analysis was carried out as previously described (Ossipova et al., 2009). Briefly, whole embryos or animal cap explants were lysed in a buffer containing 1% Triton X-100, 50 mM sodium chloride, 50 mM Tris-HCl at pH 7.6, 1 mM EDTA, 0.6 mM phenylmethylsulphonyl fluoride (PMSF), 10 mM sodium fluoride and 1 mM sodium orthovanadate. The lysates were subjected to SDS-polyacrylamide gel electrophoresis, and proteins were transferred to the PVDF membrane for immunodetection with anti-HA or anti-β-catenin antibody (1:200, Sigma, rabbit polyclonal). Chemiluminescence was captured by the ChemiDoc MP imager (BioRad).

## RESULTS

### Vangl2 is localized to the anterior edges of *Xenopus* neuroectoderm cells

**Fig. 1.**
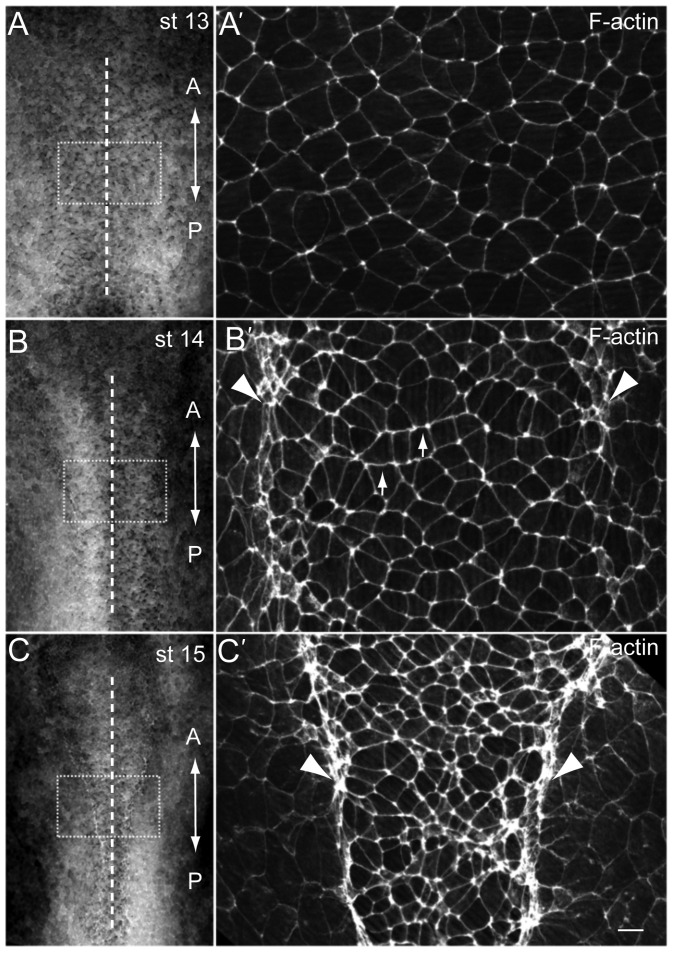
Polarized F-actin cables in the *Xenopus* neural plate. F-actin was visualized by phalloidin staining in the *Xenopus* neural plate at the beginning of neurulation. Representative explants are shown at stage 13 (st. 13, A), st. 14 (B) and st.15 (C). Boxed regions are magnified in A′–C′. Apical constriction of neural plate cells is first evident along the dorsolateral hinge regions (arrowheads), and later throughout the neural plate. F-actin cables become more pronounced in the mediolateral rather than anteroposterior orientation, forming stripes along anteroposterior cell faces (arrows). The anteroposterior (‘A–P’) axis of the neural plate is indicated. Dashed line, the neural plate midline. Scale bar, 20 µm.

After *en face* staining of the *Xenopus* neural plate with phalloidin, we were able to distinguish several morphogenetic steps of neural tissue development (Fig. 1). At the early neural plate stage (stage 13), surface epithelial cells lack significant polarization along the anteroposterior or mediolateral axes. At stage 14, apical constriction is detectable in lateral hinge regions of the neural plate. At the midline, F-actin cables become more pronounced at the cell borders perpendicular to the AP axis, forming stripes along anteroposterior cell faces. This pattern is similar to the one described for the core PCP protein Celsr1 in the chick neural tube (Nishimura et al., 2012). By stage 15, the onset of neural fold elevation is accompanied by the decrease of cell apical surfaces, as compared to the epithelial cells at the neural plate border, and by enhanced lateral hinges (Fig. 1). These events illustrate the morphological polarization of the neural plate along the anteroposterior and mediolateral axes by the onset of neural tube closure.

**Fig. 2.**
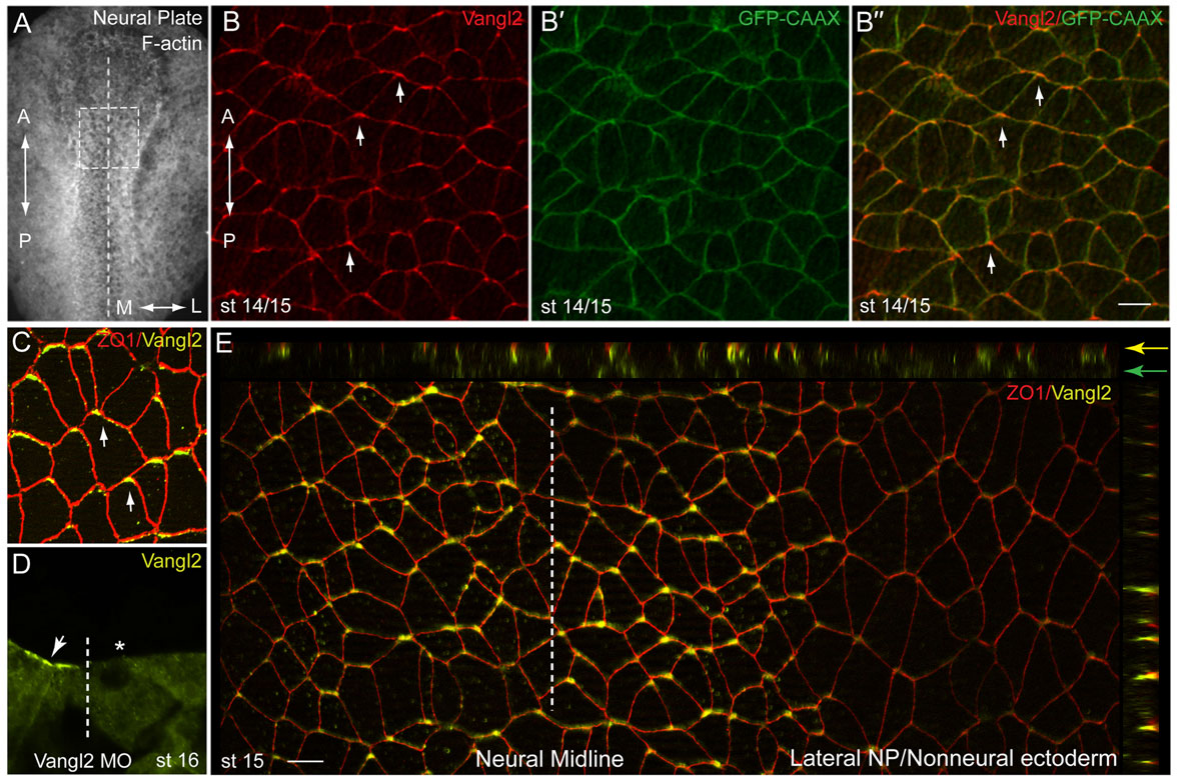
Non-homogeneous subcellular distribution of Vangl2 along the anteroposterior axis. Early embryos were injected with 100 pg of GFP-CAAX RNA to label cell boundaries. At early neural plate stage, embryos were fixed and immunostained with anti-Vangl2 and anti-GFP antibodies. *En face* view of the neural plate is shown, anterior is to the top. (A) Neural plate explant with the approximate position of the imaged area (B–B″) (boxed). Dashed line indicates the neural midline, also applies to (D,E). The anteroposterior (‘A–P’) and the mediolateral (‘M–L’) axes are shown. (B) anti-Vangl2 and (B′) anti-GFP staining (B″) Merged image. Arrows point to anterior Vangl2. (C) Double staining with anti-Vangl2 and anti-ZO1 reveals the anterior localization of Vangl2. (D) Cross-section of a neurula stage embryo injected with Vangl2 MO. Vangl2 immunostaining is reduced by the unilateral injection of Vangl2 MO (20 ng) (asterisk). Arrow points to apical Vangl2 at the uninjected side. (E) Apotome imaging of a control embryo costained with anti-Vangl2 and anti-ZO1. Z-stacks reveal apical/subapical staining of anterior Vangl2 patches at the midline (yellow arrow) as compared to the lateral/basolateral staining in non-neural ectoderm (green arrow). The midline is indicated. Scale bars, 20 µm.

The distribution of the core PCP component Vangl2 was studied in the neural plate using specific antibodies. Vangl2 immunoreactivity was largely confined to cell junctions, as expected for a transmembrane protein, but the staining was typically concentrated to the most anterior cell vertex (Fig. 2A,B). By contrast, marking cell boundaries by the expression of GFP-CAAX (Fig. 2B–B″), or co-staining with ZO1 (Fig. 2C) showed equal fluorescence intensity for all cell borders. The staining often appeared to be confined to membrane subdomains positioned near the anterior cell border (as has been verified in later experiments, see below). Lack of staining in cells depleted of Vangl2 with a specific morpholino confirmed antibody specificity (Fig. 2D). Optical sectioning and immunostaining of cryosections revealed that the enhanced Vangl2-positive areas at the neural midline were largely apical, whereas in the non-neural ectoderm Vangl2 staining was basolateral, without detectable planar polarity (Fig. 2E; supplementary material Fig. S1). The anterior polarization of Vangl2 was observed as early as stages 12.5–13 in the posterior neural plate (supplementary material Fig. S2). These findings establish Vangl2 as a new molecular marker for the anteroposterior planar cell polarity (AP-PCP) in the vertebrate neural axis.

### Vangl2 localization is directed by Prickle

**Fig. 3.**
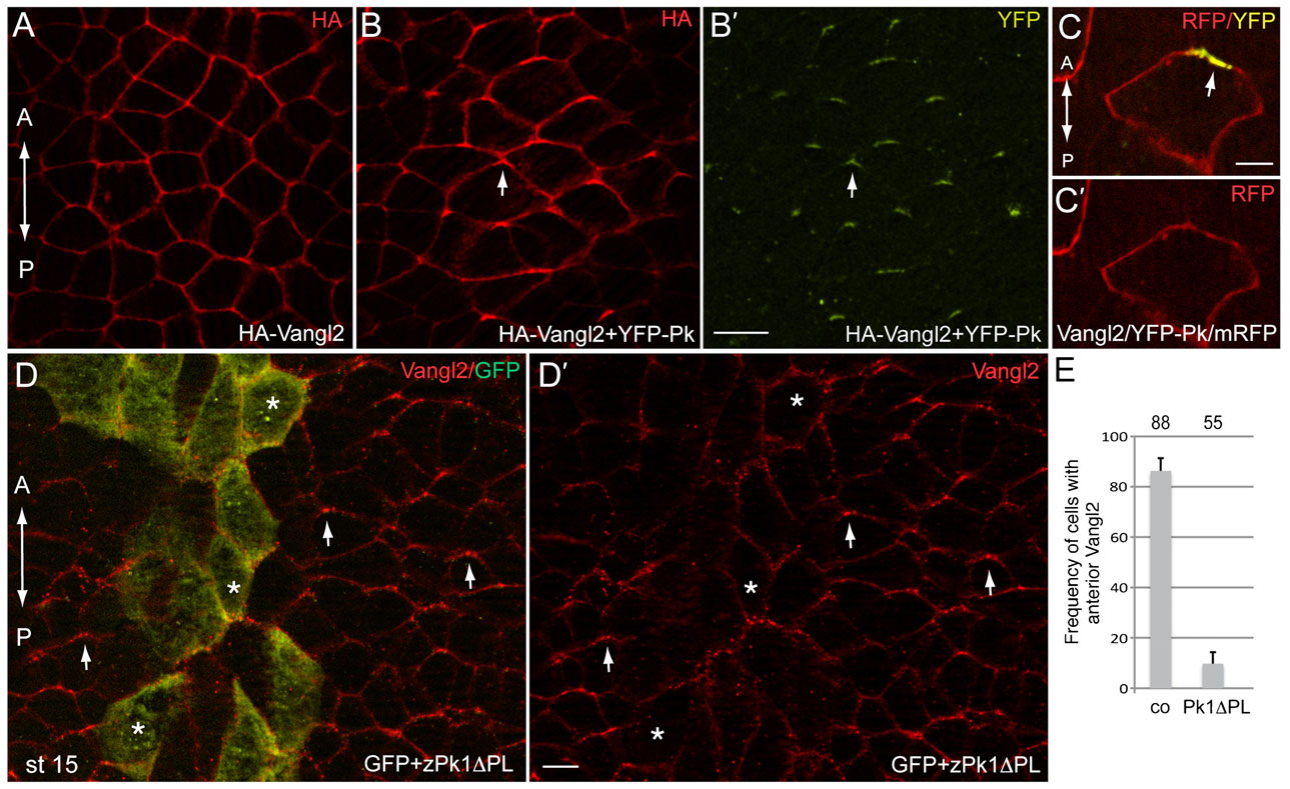
Vangl2 polarity is directed by Prickle. Early embryos were injected with HA-Vangl2 (150 pg) and YFP-Pk RNAs (100 pg) either separately or together as indicated. At early neural plate stage, embryos were fixed and immunostaned with anti-HA or anti-GFP antibodies. (A) HA-Vangl2 is homogeneously distributed at cell boundaries. (B,B′) The complex of Vangl2/Pk is polarized at the anterior end of each cell. HA-Vangl2 (B) and YFP-Pk (B′). (C,C′) The anterior localization of the Vangl2/YFP-Prickle complex in an expressing cell is visualized by YFP and membrane RFP (mRFP) epifluorescence. (D,E) Early embryos were coinjected with zPk1ΔPL RNA (1.5 to 2 ng) and GFP as lineage tracer (100 pg). (D,D′) Immunofluorescence reveals Vangl2 anterior accumulation (arrows) in the neural plate, with the exception of the cells containing zPk1ΔPL (marked by GFP, asterisks). Dorsal view of the neural plate midline is shown, anterior is to the top. (E) Quantitation of the data shown in D. See also Fig. 4C for control RNA effect. Error bars represent s.d. Co, control cells on the uninjected side. Scale bars are 20 µm in B,D, and 5 µm in C.

We next wanted to assess the distribution of exogenous Vangl2 that is expressed in early embryos by targeted mRNA microinjection. In contrast to planar-polarized endogenous Vangl2, both HA-tagged Vangl2 and CFP-Vangl2 were present at cell junctions and enriched at cell vertices in the neural plate, without an obvious anteroposterior bias in protein distribution (Fig. 3A; data not shown). A likely explanation for this discrepancy is that some PCP component is limiting in our assays. One candidate for this limiting factor is Prickle, previously shown to interact with Vangl2 (Bastock et al., 2003; Jenny et al., 2003). We assessed whether the addition of Prickle might restore the polarization of exogenous Vangl2. Indeed, coexpression of HA-Vangl2 and YFP-Pk triggered the enrichment of both proteins at the anterior end of each cell (Fig. 3B,B′). The anterior localization of the Vangl2/YFP-Pk complex was confirmed in mosaically expressing cells (Fig. 3C,C′). These observations indicate that exogenous Vangl2 does not polarize to the anterior domain on its own, but acquires this ability when in complex with Prickle.

We next tested whether the polarity of endogenous Vangl2 requires Prickle function. Since Prickle1 is a vertebrate homolog of *Drosophila* Prickle, which is expressed in the neural plate (Wallingford et al., 2002b), we constructed a variant of Prickle1, lacking the PET/LIM domain (Pk1ΔPL), which acts in the dominant negative manner (Takeuchi et al., 2003; Ossipova et al., 2015). The anterior localization of Vangl2 was disrupted in the cells expressing Pk1ΔPL, demonstrating that Prickle activity is needed for Vangl2 anterior polarization (Fig. 3D–E).

These experiments suggest that Prickle activity is both necessary and sufficient for the establishment of the Vangl2 polarity in the neural plate.

### Role of Wnt signaling in the regulation of AP-PCP at the neural plate

**Fig. 4.**
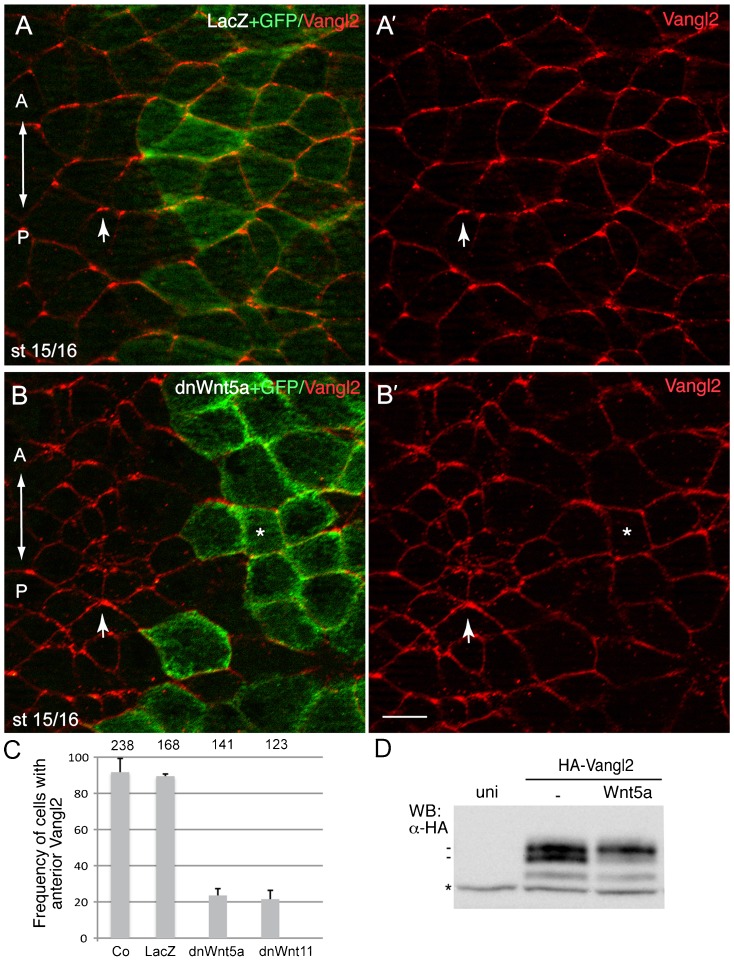
A role of Wnt signaling in establishing the Vangl2 polarity. (A,B) Early embryos were injected with DN-Wnt5a RNA (0.3 ng) and GFP RNA (0.1 ng) as a lineage tracer. Neural plate cells mosaically expressing this construct reveal lack of Vangl2 enrichment at the anterior of each cell (asterisks) as compared to non-expressing cells (arrows). A', B' are single-channel images corresponding to A and B. Control *LacZ* RNA (1.5 ng) coinjected with GFP RNA (0.1 ng) had no effect on anterior distribution of Vangl2. Dorsal view of the neural plate is shown, anterior is to the top. Scale bar, 20 µm. (C) Quantification of data from the experiments with DN-Wnt constructs showing mean frequencies of cells with anterior Vangl2±s.d. At least 5–10 embryos were examined per each treatment. Numbers of scored cells are shown on top of each bar. (D) Vangl2 is phosphorylated in response to Wnt5a in *Xenopus* ectoderm. Early embryos were injected with HA-Vangl2 mRNA (0.1 ng) together with Wnt5a RNA (0.5 ng). Cell lysates were prepared at the midgastrula stage (st. 11) for western analysis with anti-HA antibodies. Wnt5a causes HA-Vangl2 to migrate slower in whole embryo lysates (st. 11). Asterisk indicates a non-specific protein band reflecting protein loading.

The vertebrate anteroposterior axis is specified during gastrulation by secreted Wnt proteins (Hikasa and Sokol, 2013; Itoh and Sokol, 1997; Itoh et al., 1995; Kiecker and Niehrs, 2001). Several Wnt proteins including Wnt5a and Wnt11b are expressed at the posterior region of the embryo at the late gastrula stage (Hikasa and Sokol, 2013; Li et al., 2008; Walentek et al., 2013). Although PCP proteins are commonly assumed to control neurulation in response to Wnt signaling, the direct evidence for Wnt-dependent PCP signaling in the neural plate has been lacking. Due to previous reports implicating Wnt ligands in different PCP models (Gao et al., 2011; Qian et al., 2007; Wu et al., 2013), we reasoned that some ‘non-canonical’ Wnt proteins might function to establish AP-PCP in the neural plate. To test this hypothesis, presumptive neuroectoderm cells were targeted with DN-Wnt5a, a truncated Wnt5a construct previously shown to inhibit Wnt5a activity (Choi and Sokol, 2009). The anterior localization of Vangl2 was disrupted in cells expressing DN-Wnt5 RNA, but not in those expressing *LacZ* RNA (Fig. 4A–C). Similar inhibitory effect on anterior localization of Vangl2 was observed with DN-Wnt11, reported to inhibit the function of both Wnt5 and Wnt11 ligands (Tada and Smith, 2000) (Fig. 4C). This finding indicates that noncanonical Wnt signaling is indeed responsible for the observed pattern of Vangl2 localization.

### Wnt-induced Vangl2 phosphorylation in the establishment of the Vangl2 polarity

To investigate how Wnt signaling influences AP-PCP, we examined a role for Vangl2 phosphorylation that was proposed to be critical for Vangl2 localization in the mouse limb (Gao et al., 2011). Ectodermal cells stimulated with Wnt5a revealed lower mobility of Vangl2 protein in the SDS-PAGE gels (Fig. 4D). By contrast, Vangl2SA, the non-phosphorylatable form of Vangl2 (with serine to alanine substitutions at positions 5, 82 and 84), was not shifted in response to Wnt5a (Gao et al., 2011) (data not shown). These results support previous observations and suggest that Vangl2 is phosphorylated in response to Wnt5a in *Xenopus* ectoderm.

**Fig. 5.**
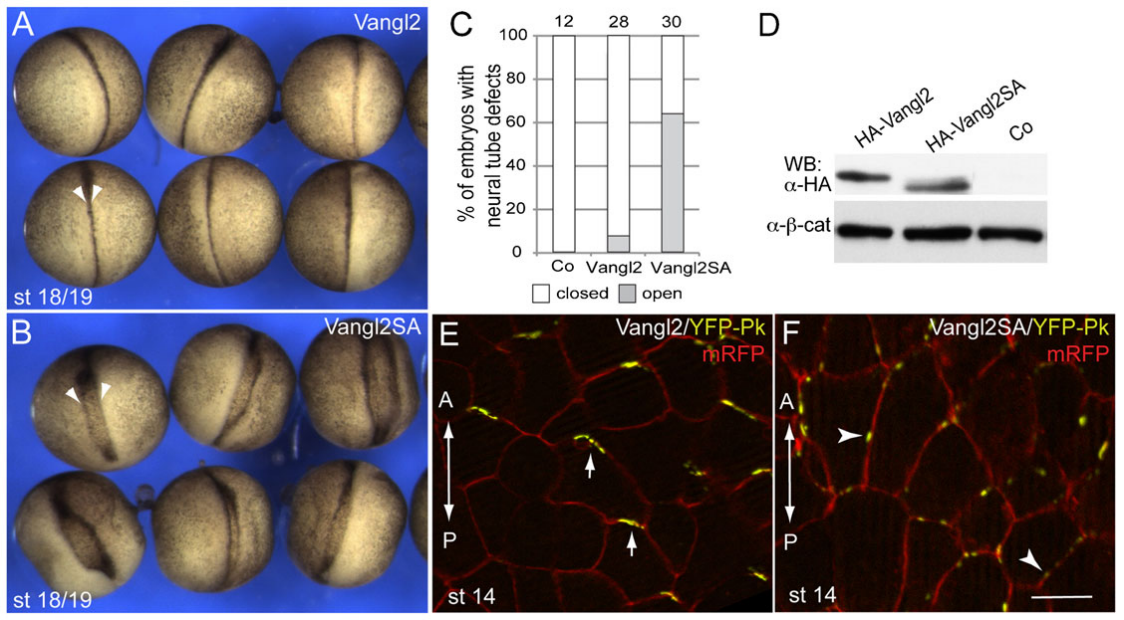
Wnt-induced Vangl2 phosphorylation in the establishment of the Vangl2 polarity. (A–C) Different activities of overexpressed Vangl2 and Vangl2SA RNAs on neural plate closure. (A) Normal neurulation in embryos overexpressing wild-type Vangl2 RNA (100 pg). (B) Neural tube defects in embryos overexpressing Vangl2SA RNA (100 pg). (C) Quantification. (D) Immunoblot analysis with anti-HA antibodies shows comparable levels of Vangl2 constructs. Anti-β-catenin antibodies reveal protein loading. Note faster migration of non-phosphorylatable Vangl2. The image of lane containing uninfected embryo lysate (Co) is obtained from the same gel. (E,F) Phosphorylation of Vangl2 is critical for the establishment of AP-PCP. YFP-Pk mRNA (50–100 pg) was unilaterally coinjected with HA-Vangl2 RNA or HA-Vangl2SA RNAs (100 pg each) at the 8-cell stage. mRFP RNA (100 pg) is a lineage tracer. AP-PCP is assessed by the recruitment of Pk to anterior (arrows) or lateral (arrowheads) cell edges near the neural midline (st 14/15) in the presence of HA-Vangl2 or HA-Vangl2SA. Scale bar, 20 µm.

The activities of wild-type Vangl2 and Vangl2SA were compared in gain-of-function assays. We observed that the Vangl2SA mutant behaved in a dominant interfering manner, causing pronounced neural tube defects as compared to wild-type Vangl2 (Fig. 5A–C). Both proteins were expressed at similar levels in these experiments (Fig. 5D). These findings further indicate the significance of Vangl2 phosphorylation for neural tube closure.

We next compared the ability of Vangl2 and Vangl2SA to polarize in the neural plate after coexpression of Prickle (YFP-Pk). Although YFP-Pk formed complexes with both wild-type and mutant Vangl2, the complex of YFP-Pk with Vangl2SA was unable to polarize in the neural plate (Fig. 5E,F). These experiments suggest that AP-PCP is established via Wnt-dependent Vangl2 phosphorylation.

### Feedback regulation of Vangl2 localization by Myosin II

Myosin II is a multifunctional motor protein that controls cell behavior in a variety of morphogenetic processes (Bertet et al., 2004; Heisenberg and Bellaïche, 2013; Vicente-Manzanares et al., 2009). Although mechanical forces initiated by cell flow were proposed to regulate planar cell polarity in the *Drosophila* embryo (Adler, 2012; Aigouy et al., 2010), the role of Myosin II in the establishment of core PCP protein localization has not been addressed. Therefore, we wanted to know whether, in addition to Wnt signaling, Myosin II activity is critical for Vangl2 polarity generation.

**Fig. 6.**
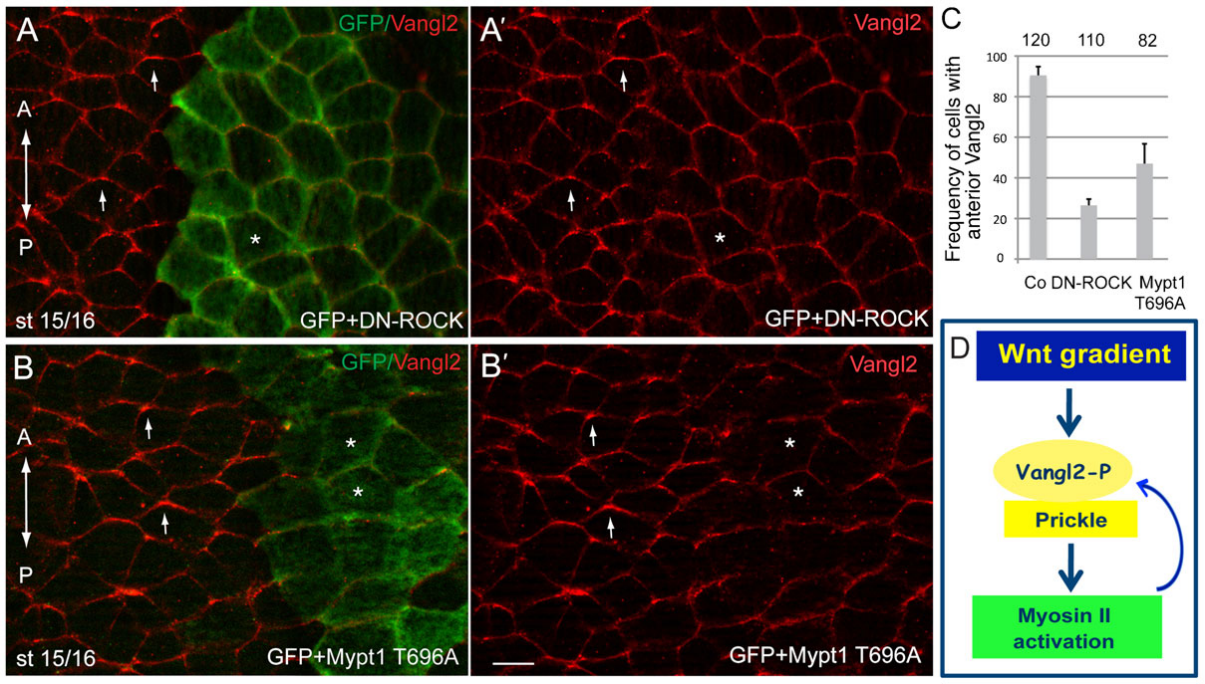
Feedback regulation of AP-PCP by the ROCK/Myosin II pathway. (A,B) Eight cell embryos were unilaterally coinjected with the lineage tracer (GFP RNA, 100 pg) and DN-ROCK RNA (100 pg, A) or Mypt1T696A RNA (100 pg, B) and Vangl2 polarity was assessed by immunostaining at the neural plate stage. Both the interference with ROCK signaling (A,A′) and the dephosphorylation of Myosin II light chain by the myosin phosphatase Mypt1 (B, B′) inhibit Vangl2 polarity. Arrows point to polarized Vangl2 staining. Asterisks indicate treated cells marked by GFP with non-polarized Vangl2. (C) Quantification of the data shown in A,B. Error bars represent s.d. (D) Model of AP-PCP. Wnt activity gradient leads to the polarization of the Vangl2/Pk complex to the anterior cell domain. Myosin II activity, a putative target of PCP signaling, mediates feedback regulation of core PCP protein localization. Scale bar, 20 µm.

We first interfered with Rho GTPase-associated protein kinase (ROCK) that modulates Myosin II activity by phosphorylating regulatory Myosin II light chain (MLC) and MLC phosphatase (Kimura et al., 1996; Vicente-Manzanares et al., 2009). ROCK functions downstream of core PCP proteins to regulate planar polarity in the *Drosophila* wing and the eye (Winter et al., 2001) and is regulated by Wnt ligands in zebrafish embryos (Marlow et al., 2002). Neural plate cells expressing DN-ROCK failed to establish Vangl2 polarity, indicating that ROCK is involved in this process upstream or parallel to core PCP proteins (Fig. 6A,A′). Using an alternative approach, we inhibited MLC phosphorylation by the constitutively active myosin-binding subunit of myosin II phosphatase Mypt1 (Weiser et al., 2009). In cells with high levels of Mypt1 activity, Vangl2 was not polarized (Fig. 6B,B′). Together, our observations support a model, in which Myosin II is not only a target of PCP signaling, but also generates mechanical forces contributing to core PCP protein polarization (Fig. 6C,D).

## DISCUSSION

Our study revealed the polarized distribution of endogenous Vangl2 to the anterior domain of each cell in the *Xenopus* neural plate. Further analysis demonstrated that this polarity requires the formation of the complex between Vangl2 and Prickle and Wnt-dependent phosphorylation of Vangl2. One could envisage several models for the generation of AP-PCP in the neural plate. First, the anterior Vangl2/Pk complex might derive from the initial animal-vegetal polarity of the egg. This possibility is not likely, as the polarity of Vangl2 in the plane of the tissue is not detectable at the blastula or early gastrula stages (data not shown). Second, AP-PCP may reflect cell behavior and cell shape changes in the neural plate, as proposed for the PCP in the *Drosophila* embryo (Aigouy et al., 2010). Consistent with this model, the majority of cells at the neural plate midline are displaced towards the anterior end of the embryo during neurulation (Burnside and Jacobson, 1968). Third, our finding that interference with Wnt5 and Wnt11 signaling disrupts Vangl2 polarization suggests that AP-PCP is a consequence of Wnt signaling. Supporting this hypothesis, several Wnt genes are expressed at the posterior end of the embryo during gastrulation (Hikasa and Sokol, 2013; Li et al., 2008; Wolda et al., 1993) and may produce a gradient of signaling activity. Consistent with these reports, we observed that Vangl2 polarization is first detected in early neurulae at the posterior neural plate (data not shown). These findings suggest that Vangl2 polarity may be established by a gradient of Wnt activity.

The mechanistic connection between AP-PCP and neural tube closure remains to be established. The enrichment of PCP proteins only at the anteroposterior faces of the neighboring cells was suggested to lead to actomyosin-dependent constriction of PCP-protein-containing cell junctions resulting in neural plate bending (Nishimura et al., 2012). Also, PCP- and Septin-mediated restriction of actin diffusion were proposed to stimulate mediolateral intercalation of mesodermal cells (Shindo and Wallingford, 2014). Alternatively, the mediolateral localization of Rab11 and Diversin were hypothesized to drive neural plate closure by orienting apical constriction at the lateral hinges towards the midline (Ossipova et al., 2014). Our current analysis of the neural plate midline is consistent with Vangl2 influencing actomyosin dynamics at the anterior cell edges, leading to more efficient apical constriction at the midline. Indeed, Myosin II light chain was no longer phosphorylated at the midline of cells depleted of Vangl2 (Ossipova et al., 2014). The anterior polarization of Vangl2 at the midline (this study) and the medial polarization of Diversin in the lateral neural plate (Ossipova et al., 2014) suggests the existence of at least two signaling pathways that act orthogonally to coordinate neural tube closure. Notably, in the *Drosophila* wing, the two independently operating Ds/Fj and Fz/Vang signaling modules are known to function along the same proximal-distal axis (Casal et al., 2006; Devenport, 2014). Additional studies are warranted to evaluate the molecular composition and the interactions between the anteroposterior and the mediolateral PCP systems.

PCP proteins appear to regulate cell shape and junction remodeling by affecting basic cellular processes, including vesicular trafficking (Classen et al., 2005; Devenport, 2014; Gray et al., 2009; Ossipova et al., 2014), actomyosin and microtubular organization (Nagaoka et al., 2014a; Nagaoka et al., 2014b; Olofsson et al., 2014; Sepich et al., 2011; Tatin et al., 2013; Vladar et al., 2009; Vladar et al., 2012). Myosin II, a key cellular mechanosensor and mechanotransducer, and its upstream regulator ROCK (Fernandez-Gonzalez et al., 2009; Lecuit et al., 2011) have been argued to function in the Drosophila wing and eye PCP downstream of Frizzled and Dishevelled (Winter et al., 2001). Although this view is consistent with the role of ROCK/Myosin II pathway in convergent extension movements in zebrafish and *Xenopus* embryos (Marlow et al., 2002; Rolo et al., 2009; Skoglund et al., 2008), the effects of ROCK or Myosin II modulation on the localization of core PCP proteins have not been assessed. Our results indicate that both ROCK and Myosin II are critical for the establishment of Vangl2 polarity, supporting previous studies (Blair et al., 2006; Mahaffey et al., 2013; Yan et al., 2009). The observed regulatory feedback reinforces the critical role of mechanical forces in PCP, which is needed to maintain the robustness of morphogenetic processes in embryonic development.

Studies of vertebrate PCP proteins reveal their roles in many morphogenetic processes, including asymmetric cell division (Ciruna et al., 2006; Lake and Sokol, 2009; Ségalen et al., 2010), collective cell migration (Gray et al., 2011; Keller, 2002; Sokol, 1996; Tada and Heisenberg, 2012; Wallingford et al., 2002a) and apical constriction (Ossipova et al., 2015). These expanding functions might reflect the diversity of morphogenetic strategies used by chordate embryos to acquire the same basic body plan (Kirschner and Gerhart, 1998; Shook and Keller, 2008). Indeed, the different roles of Vangl2 in asymmetric cell division in zebrafish (Ciruna et al., 2006) and apical constriction in *Xenopus* (Ossipova et al., 2015) are likely due to species-specific differences in morphogenesis. Despite these variations, AP-PCP is remarkably conserved in the mouse skin and the node (Antic et al., 2010; Borovina et al., 2010; Devenport and Fuchs, 2008; Hashimoto et al., 2010), the ascidian notochord (Jiang et al., 2005) and the chick neural plate (Nishimura et al., 2012). Future studies are needed to determine whether AP-PCP reflects the conservation of Wnt signaling activity along the anteroposterior body axis in different models (Hikasa and Sokol, 2013; Itoh and Sokol, 1997; Kiecker and Niehrs, 2001).

## Supplementary Material

Supplementary Material
