# Supplementary material for: Planar polarization of Vangl2 in the vertebrate neural plate is controlled by Wnt and Myosin II signaling
Source: Biol Open. 2015 Apr 24;4(6):722–30. doi: 10.1242/bio.201511676 (PMC4467192; doi:10.1242/bio.201511676)
Supplement: Supplementary Material [file supp_4_6_722__index.html]

Supplementary Material 

# Planar polarization of Vangl2 in the vertebrate neural plate is controlled by Wnt and Myosin II signaling

## bio.201511676 Supplementary Material

Olga Ossipova et al. doi: 10.1242/bio.201511676

**Files in this Data Supplement:**

- Supplementary Material - Olga Ossipova et al. doi: 10.1242/bio.201511676
