# Supplementary material for: Planar polarization of Vangl2 in the vertebrate neural plate is controlled by Wnt and Myosin II signaling
Source: Biol Open. 2015 Apr 24;4(6):722–30. doi: 10.1242/bio.201511676 (PMC4467192; doi:10.1242/bio.201511676)
Supplement: Supplementary Material [file bio.201511676_bio.201511676-s1.pdf]

Biology Open (2015) 000, 1–9 doi:10.1242/bio.201511676

Supplementary Material
Olga Ossipova et al. doi: 10.1242/bio.201511676

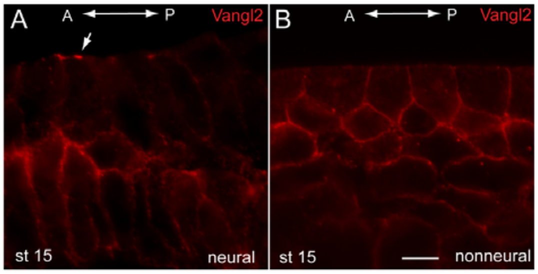

Fig. S1. Different localization of Vangl2 at the neural midline and in non-neural ectoderm. Sagittal sections of stage 15 embryos immunostained with anti-Vangl2 antibody. Anteroposterior (‘A–P’) axis is indicated. Apical surface is at the top. (A) Vangl2 is enriched at the apical surface of cells at the neural midline (arrow). (B) Basolateral localization of Vangl2 at the cell junctions of non-neural ectoderm. Scale bar, 20 µm.

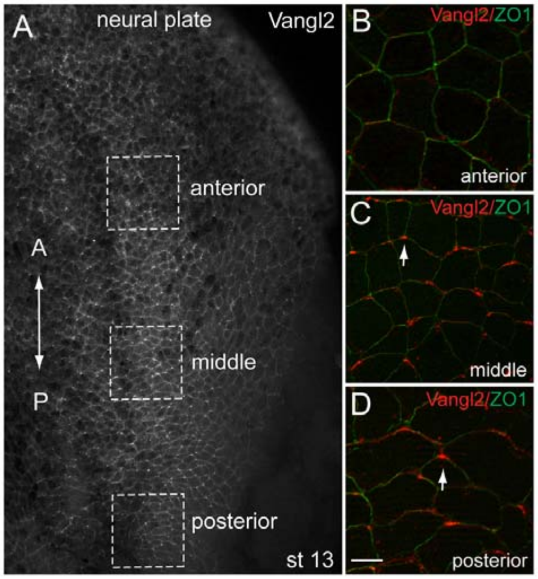

Fig. S2. Polarized distribution of Vangl2 along the anteroposterior axis in the early neural plate. Apotome imaging of an early neurula embryo (st 13) costained with anti-Vangl2 and anti-ZO1. *En face* view of the neural plate is shown, anterior is to the top. (A) Neural plate explant with approximate positions of the imaged areas (boxed). The anteroposterior (‘A–P’) axis is indicated. (B–D) High magnification views of boxed regions shown in A. Vangl2 is barely detectable in the anterior region of the neural plate (B). Arrows point to polarized Vangl2 in the middle (C) and the posterior (D) portions of the neural plate. Scale bar, 20 µm.
